# Modelling the Spatial Behaviour of a Tropical Tuna Purse Seine Fleet

**DOI:** 10.1371/journal.pone.0114037

**Published:** 2014-12-02

**Authors:** Tim K. Davies, Chris C. Mees, E. J. Milner-Gulland

**Affiliations:** 1 MRAG Ltd, London, United Kingdom; 2 Imperial College London, London, United Kingdom; UC Santa Cruz Department of Ecology and Evolutionary Biology, United States of America

## Abstract

Industrial tuna fisheries operate in the Indian, Atlantic and Pacific Oceans, but concerns over sustainability and environmental impacts of these fisheries have resulted in increased scrutiny of how they are managed. An important but often overlooked factor in the success or failure of tuna fisheries management is the behaviour of fishers and fishing fleets. Uncertainty in how a fishing fleet will respond to management or other influences can be reduced by anticipating fleet behaviour, although to date there has been little research directed at understanding and anticipating the human dimension of tuna fisheries. The aim of this study was to address gaps in knowledge of the behaviour of tuna fleets, using the Indian Ocean tropical tuna purse seine fishery as a case study. We use statistical modelling to examine the factors that influence the spatial behaviour of the purse seine fleet at broad spatiotemporal scales. This analysis reveals very high consistency between years in the use of seasonal fishing grounds by the fleet, as well as a forcing influence of biophysical ocean conditions on the distribution of fishing effort. These findings suggest strong inertia in the spatial behaviour of the fleet, which has important implications for predicting the response of the fleet to natural events or management measures (e.g., spatial closures).

## Introduction

Fisheries for tropical and temperate tunas operate on an industrial scale in the Indian, Atlantic and Pacific Oceans, landing more than 4.2 million tonnes in 2011. Of the 23 commercially exploited tuna stocks worldwide, 9 are currently considered to be in an overfished state [1]. Overcapacity in tuna fleets, both in terms of the number of vessels and their ability to catch and store fish, is a serious concern in the conservation and management of tuna stocks, resulting in overfishing and significant economic waste [2]. These concerns over sustainability and impact have prompted a critical look at the way in which tuna fisheries exploit the resource, in particular the techniques used and the design of fishing gears employed to catch tunas [3], [4]. There has also been increasing scrutiny of how fisheries are managed, with many tuna regional fisheries management organisation (tRFMOs) criticised for a notable absence of ‘modern’ philosophies such as the precautionary and ecosystem-based approaches to management [5], [6].

Failures in fisheries management can result not only from insufficient understanding of the biological dynamics of an exploited resource, but also from uncertainty in the actions of fishers [7]–[10]. The ability to anticipate fishers' behaviour has become an increasingly important focus of research in fisheries science [10]–[12], although to date there has been little research directed at understanding behavioural dynamics in tuna fisheries. As a result, there remain many uncertainties that have the potential to undermine management. For instance, how will a fleet respond to the implementation of a management measure, such as a spatial closure or a gear restriction; or a change in political and economic conditions, such as an increase in piracy or fuel costs; or perturbations in environmental conditions, such as anomalous climatic events: and how will the response ultimately affect management outcomes? An improved understanding of fishers' behaviour is a necessary first step in answering these questions.

The spatial behaviour of fishers can be considered at many different scales. In the short term, the behaviour of fishers might be considered at a fine scale, for example the day-to-day movement of an individual between reefs or banks. These fine scale behaviours are usually directed at meeting an immediate challenge, such as maximising the day's catch, and might be influenced primarily by personal experience and the information available [13], [14]. In the longer term, it may be more relevant to consider aggregate behaviours at a broad scale, such as the seasonal movement of a fishing fleet (Table 1). These fleet-level behaviours are the product of common strategies or coordinated behaviours that are determined by broad environmental or company-level influences [7], [9]. Furthermore, some aggregate behaviours may not necessarily be the result of short term planning by individuals or firms but instead emerge through cooperation or competitive interactions within the fleet [7]–[9].

**Table 1:** Varying units and spatiotemporal scales at which the behaviours of fishers may be observed.

Scale	Short term, fine scale	Long term, broad scale
Decision unit	Individual fisher	Fishing fleet
Movements	e.g., tactical fishing manoeuvres, moving between local grounds	e.g., seasonal movement, fishing along closed area boundaries
Influences	e.g., skill and experience, vessel characteristics, fishing preferences	e.g., seasonal environmental processes, company strategy, intra-fleet interactions

Several modelling approaches have been developed to explain and predict the spatial behaviour of fishers, with discrete choice models being particularly popular in the fisheries economics literature [12]. An attractive feature of some discrete choice models (e.g. mixed logit models) is that they do not assume homogeneity in the decision making of individuals, which is particularly useful when predicting behaviour in fisheries where the incentives and constraints that determine behaviour vary markedly between fishers [15], [16]. However, a potential shortcoming of this modelling approach is that large panel datasets are required to describe the range of choices faced by individuals, which can become huge and therefore computationally demanding when fine spatial scales are considered. In an alternative approach, a number of conceptual models have been developed to examine the drivers of fleet-level spatial behaviour, which by definition do not consider discrete choices, and consequently may be more appropriate for modelling the general movement of a fleet in space. However, these models, which move effort between locations according to some index of suitability, necessarily require preconceptions as to what constitutes an ‘attractive’ location, for example the availability of the resource or competition from other vessels in the fleet [17]–[19].

An alternative modelling approach is found in the ecology and conservation science literature, where a number of statistical modelling approaches have been developed for investigating the spatiotemporal distribution of a species in a landscape [20]. Within the field of species distribution modelling, a subset of regression-based models have been used to characterise the distribution of a species or activity, explain the functional relationship between an organism and the environment, and to generate insight into a species' behavioural ecology or evolutionary history (see [21] for a review).

The aim of this study was to improve understanding of the spatial behaviour of tropical tuna fleets, using the Indian Ocean tuna purse seine fishery as a case study. Our analysis was informed by two *a priori* hypotheses of the factors that influence the distribution of fishing effort, based on practical knowledge of the fishery and the wider literature on fisher behaviour. Firstly, the distribution of tropical tunas is influenced by the biophysical ocean environment [22]–[24], and purse seine skippers use satellite-derived information on a number of key environmental conditions to identify promising fishing locations in the short term (J. J. Areso, Spanish fleet representative, pers. comm., June 2011). We therefore asked whether environmental conditions influence the distribution of fishing effort at broad spatiotemporal scales. Secondly, many previous studies of decision making by fishers have demonstrated a strong link between past and future behaviour, termed variously as habit, tradition or inertia [15], [16], [25]–[27]. We therefore also examined the relationship between the past and future behaviour of the fleet, and discuss the implications of this in anticipating the behavioural response of the fleet. Focus was placed on the behaviour of the fleet at broad spatiotemporal scales, rather than on the behaviour of individual vessels, as, from a management perspective, this was considered to be the relevant scale in regards to anticipating broad changes in fleet dynamics.

## Methods

### Description of the fishery

The tropical tuna purse seine fishery targets three main tuna species (skipjack *Katsuwonus pelamis*, yellowfin *Thunnus albacares*, and bigeye tuna *T. obesus*) across the majority of the western Indian Ocean throughout the year [28]. Tunas are targeted as free-swimming schools (free schools) or in association with floating objects, such as natural debris or purpose-built drifting fish aggregating devices (FADs) [29]. Purse seine vessels are equipped with sophisticated navigation and fish-finding technology, and although capable of extended fishing trips lasting several weeks, vessels must return to port regularly to land or tranship catch and resupply. The size of the active fleet fluctuates with the perceived availability of fishing opportunities in the Indian Ocean and 34–52 vessels per year have operated in the fishery since 2000. The fleet is dominated by Spanish and French owned-and-operated vessels.. The port of Victoria, Seychelles, is the main port used by the fleet as its position in the geographic centre of the region allows skippers to minimise steaming time and maximise fishing days [30].

**Figure 1:**
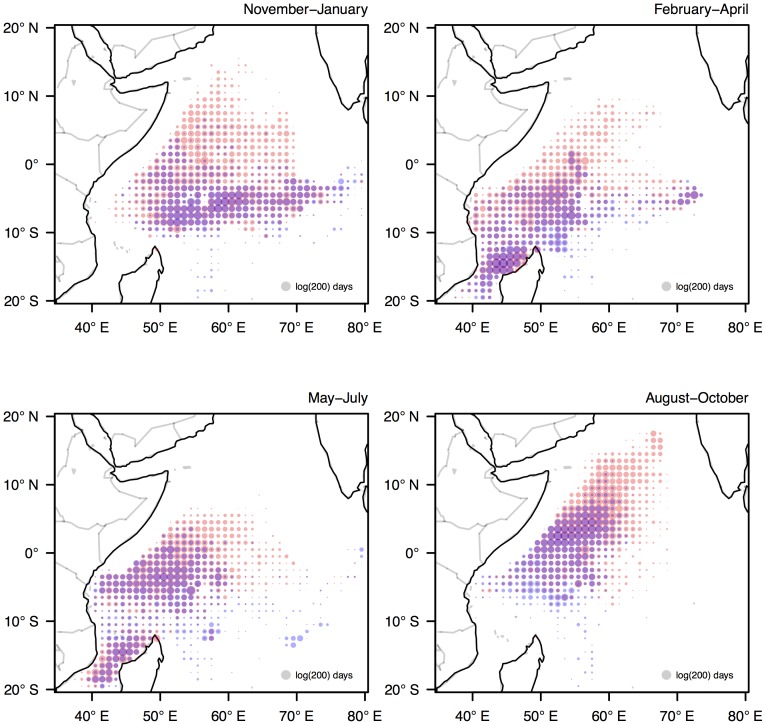
Seasonal patterns of fishing effort by the Spanish (red) and French (blue) flagged vessels in the western Indian Ocean in each of four fishing seasons: November-January and February-April (northeast monsoon), and May-July and August-October (southwest monsoon). Circle size shows the total log fishing days allocated into each grid cell (1°×1°) in each season during 2007–2011.

At broad spatiotemporal scales the spatial behaviour of the fleet is characterised by seasonality in the use of fishing grounds, and the clustering of fishing effort in space. Throughout the year the fleet transitions between three main fishing grounds: the northwest grounds (associated with the practice of fishing around floating objects), the central equatorial grounds (associated with the practice of fishing on free schools) and the southwest grounds (associated with a mixture of both fishing practices). The timing of the movement between these grounds coincides approximately with the southwest (boreal summer) and northeast (boreal winter) monsoons (Fig. 1). Whilst the use of these seasonal grounds is similar between the French and Spanish fleet components, the latter tends to fish in the northwest grounds for a greater part of the year due to the FAD-centric fishing strategies employed by some Spanish fishing companies [28]. The clustering of fishing effort is partly due to the aggregated nature of the fisheries data, as during the course of month, a single vessel is likely to report effort in adjacent grid cells. This clustering is further amplified by the high levels of cooperation and information sharing in the fishery, which results in skippers fishing in close vicinity to others. However, as cooperation occurs mainly between vessels allied by fishing company or flag nationalities, this clustering of effort is mainly observed in the allocation of effort of the respective fleet components (see Fig. 1).

### Data

The behaviour of the fleet was considered at the spatial resolution of 1° latitude/longitude grid cells and the temporal resolution of one month. Purse seine fishing data were available from the Indian Ocean Tuna Commission (IOTC; www.iotc.org, downloaded Sept 2012). Data were disaggregated by flag nationality to distinguish between the French, including vessels flagged to French Territories (33.7% registered vessels), and Spanish, including Spanish-owned vessels flagged to Seychelles (53.1% registered vessels), components of the fleet as these had a consistent presence in the western Indian Ocean during the period analysed (2007–2011).

Data were obtained for four aspects of the biophysical ocean environment relevant to purse seine fishing; sea surface chlorophyll-*a* (SSC; mg/m^3^), sea surface temperature (SST; °C), sea level anomaly (SLA; cm) and wind speed (m/s). All environmental variables were downloaded in 8-day intervals but averaged by month to correspond with fisheries data. This averaging inevitably smoothed over short term oceanographic features that may influence the fine scale allocation of effort, although basin scale environmental gradients were preserved. Data for SSC and SST were obtained from measurements produced by the MODIS sensor, made available for download by the Distributed Active Archive Centre of the Goddard Space Flight Centre/NASA (available at http://disc.sci.gsfc.nasa.gov, downloaded August 2012). SSC was log transformed to improve the spread of skewed values. SLA data were obtained from information collected by the TOPEX and Poseidon altimeters, made available for download by Aviso (available at http://www.aviso.oceanobs.com, downloaded August 2012). Data on wind speed were collected by Envisat and made available for download by MyOcean (available at http://www.myocean.eu, downloaded August 2012).

### Statistical modelling

A series of models was fitted to the data. The response variable was binary, indicating whether or not fishing effort was observed in a location in a given month. Nine explanatory variables were chosen to construct models, based on an understanding of the tools, techniques and fishing practices used by skippers to find tunas, although only variables that were relevant in explaining the behaviour of the fleet at a broad spatiotemporal scale considered (Table 2).

**Table 2:** Summary of the explanatory variables considered in the models, their predicted effect on effort allocation into an area and data sources.

Variable	Description	Range/units	Data source
**Categorical variables**			
Year	Calendar year	2007–2011	-
Season	Quarterly period; February-April, May-July, August-October, November-January	1-4	-
Flag	Flag nationality of reported effort	France/Spain	IOTC
Past use	Frequency with which the location was fished in the same month in the previous five years by vessels of the same flag nationality	0–5	IOTC
**Continuous variables**			
SSC	Log-transformed sea surface chlorophyll-*a*; proxy for primary productivity	0.02–25.8 mg/m^3^	MODIS
SST	Sea surface temperature	22–32°C	MODIS
SLA	Sea level anomaly; proxy for thermocline depth	−36–50 cm	Topex/Poseidon
Wind speed	Wind speed at 10 m above the sea surface	0–15 m/s	Envisat
Distance	Distance from the port of Victoria, Seychelles (calculated using the Spherical Law of Cosines)	0–3,000 km	-

All variables were aggregated at monthly intervals and at a spatial resolution of 1° latitude/longitude.

Four variables described the biophysical characteristics of the location; oceanographic conditions (*SST*, sea surface temperature; *SSC*, sea surface chlorophyll-*a* concentration; *SLA*, height of sea level anomaly) and meteorological conditions (*wind*, wind speed over the sea surface). In most studies of fleet dynamics, expected revenue (e.g. estimated by past catch rates or value) is an expected driver of behavior. In this case, given high spatial variability in catches from one year to the next, environmental conditions were instead used as an approximate proxy for expected revenue (i.e. the expected distribution of tunas). The past behaviour of the fleet was described as the frequency that fishing effort reported by vessels of the same flag nationality was observed in the location in the same month in the previous five years, thus taking into account seasonality in the use of fishing grounds. The variable *distance* described the position of the location relative to the port of Victoria, which for simplicity was taken to be the main port used by the fleet. In addition to the main effects, the variables *year*, *month* and *flag* were included to account for possible temporal variation in the spatial footprint of the fleet.

One factor that was expected to have an important influence on fleet behaviour, but was not included in this analysis, was the location of FADs. Fishing using FADs has become the dominant fishing practice in tuna purse seine fisheries worldwide, and in the Indian Ocean the use of FADs has been important in shaping spatiotemporal fishing patterns (see [28] for a review). The position and density of FADs is known to the fishing industry, but unfortunately it was not possible in this study to access these data.

### Autocorrelation

In this study there was a strong possibility of both temporal and spatial autocorrelation in the model residuals. Temporal autocorrelation was expected due to the strong seasonal patterns observed in the movement of the fleet, and spatial autocorrelation was expected due to the clustering of reported fishing effort in space. Such autocorrelation violates one of the key assumptions of the statistical model used here: that residuals are independently and identically distributed (i.i.d.). The violation of this assumption may bias parameter estimates and can increase type I error rates (e.g. wrongly rejecting a null hypothesis of no effect).

Correlation plots were used to visually test for the presence of spatial and temporal autocorrelation in the residuals of a model fitted with all predictor variables. Autocorrelation function plots showed no significant temporal autocorrelation, and so no further action was taken. However, correlograms indicated moderate spatial autocorrelation to a lag distance of up to ∼5 degrees. A number of approach have been described to deal with spatial autocorrelation in regression modelling, including the use of autocovariates or spatial eigenvector mapping [31], both of which were trialled in this study. However, these approaches introduced additional non-trivial issues that affected the fitting or interpretation of model results, and eventually a decision was made to proceed without attempting to address spatial autocorrelation.

### Model structure

A candidate set of generalised additive models (GAMs) was chosen *a priori* and fitted to the data using R 2.15 (R Development Core Team 2012) using the package *mgcv* [32]. GAMs were chosen over generalised linear models due to their ability to deal with non-linear relationships between the response and explanatory variables, which was useful for examining the potentially complicated effect of the environmental variables. Smooth functions were used to fit to the variables *SSC*, *SST*, *SLA, wind* and *distance*. Penalized cubic regression splines were used for computing efficiency due to the very large number of observation in the data. The degrees of freedom (or ‘wiggliness’) of the smooth functions was determined for each explanatory variable as part of the model fitting process, removing the subjectivity of manually determining knot locations [32]. The Akaike information criterion (AIC) was used to rank and assign support for the competing candidate models. This selection criterion uses maximum likelihood scores as a measure of how well the model fits the data, taking into account model parsimony.

The data were split randomly into a training dataset (90%) and a validation dataset (10%), with the latter used to evaluate the predictive accuracy of the models using the area under the Receiver Operating Characteristic curve (AUC), where a score of 0.5 indicates that model accuracy is no better than random and a score of 1 indicates perfect discrimination [33]. Average predictive comparisons were used to examine the effect size of the explanatory variables. These were calculated by comparing the mean predicted response from two modified datasets in which a focal variable was fixed at its alternative values, with all other explanatory variables left unaltered [34].

## Results

Model selection resulted in a single model containing 100% of the AIC weight, indicating a high degree of model selection certainty [35]. This AIC-best model contained all nine explanatory variables. Predictions from the best model corresponded well with the observed distribution of effort in the validation dataset (AUC  = 0.868), indicating that the model could predict the spatial behaviour of the fleet with reasonable accuracy.

**Figure 2:**
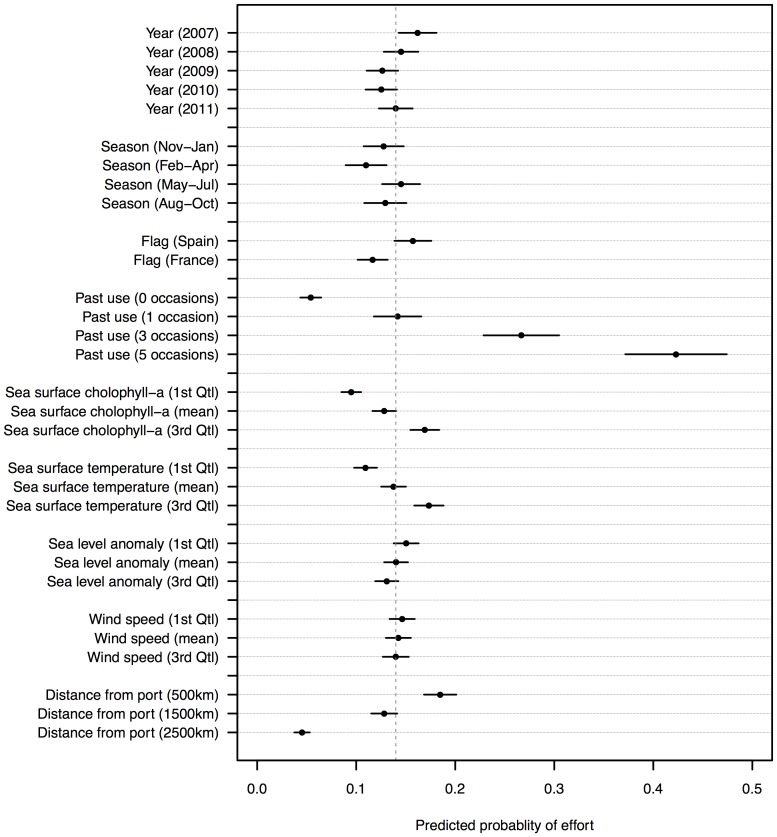
Average predicted comparison from the AIC-best model illustrating the effect of the categorical explanatory variables on the probability of observing effort in a location. The dashed vertical line indicates the predicted overall mean probability. Heavy horizontal lines through each point indicate approximate 95% confidence intervals. Note the truncated x-axis. See Table 2 for descriptions of the explanatory variables.

Average predictive comparisons illustrating the magnitude of effect of each of the predictor variables on the response are shown in Fig. 2. The variable *past use* had the largest effect on the probability of observing fishing effort in a location, indicating that fishing was increasingly more likely to be observed in locations that had been visited more frequently in the same month in previous years by vessels of the same flag nationality. Taking into account the influence of all other variables, there was only a 5.4% mean chance of observing fishing in a location that had never been visited in the recent past, whereas fishing effort was on average 36.9% more likely to be observed in a location that had been visited consistently in the previous five years.

**Figure 3:**
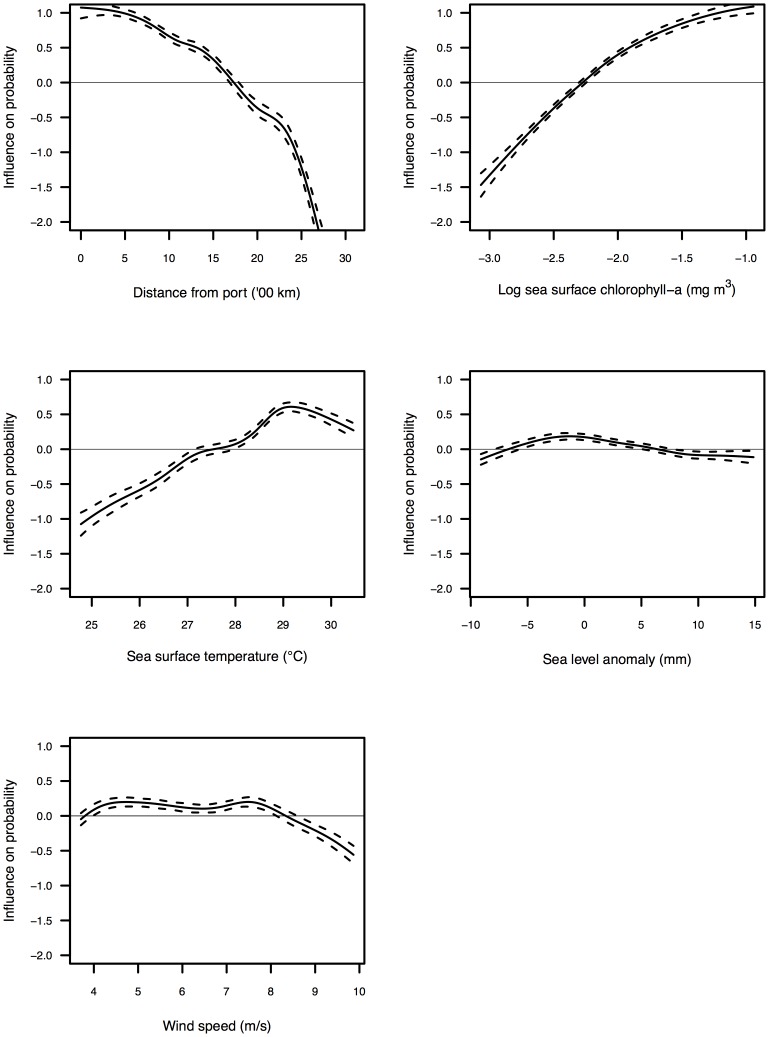
Additive components of the GAM showing the influence of the environmental variables on the probability of effort being observed in a location. The dashed lines show the standard errors. To improve interpretation, the x-axis of each panel is trimmed to show only the middle 90% of the observation. See Table 2 for a description of the explanatory variables.

The distance of a location from the port of Victoria also had an important effect on the spatial behaviour of the fleet, with fishing 13.9% less likely to be observed in a location 2,500 km from port than a location 500 km from port. This relationship between *distance* and the response was negatively exponential, with the positive influence of distance initially deteriorating gradually but becoming increasingly negative beyond 1,500 km from Victoria (Fig. 3).

The variables *SSC* and *SST* had positive effects on the probability of fishing being observed in a location, indicating that in general the fleet was more likely to fish in warmer, more biologically productive waters. For both variables the functional relationship with the response was linear throughout low and mid-range values, but at high values the positive influence either flattened out or, in the case of SST, diminished (Fig. 3). Average predictive comparisons indicated that for both variables the magnitude of effect on the response was reasonably small (Fig. 2). For example, fishing was, on average, just 3.3% more likely to be observed in a location with a mid-level SSC concentration (0.11 mg m^3^, global mean) than in a location with very a low SSC concentration (0.08 mg m^3^, 1^st^ quartile). Similarly, fishing was only 2.8% more likely to be observed in a location with a mid-level SST (28.1°C, global mean) than in a location with a relatively low SST (26.6°C, 1^st^ quartile).

The variables *SLA* and *wind* had negative effects on the probability of fishing being observed in a location, although in both cases the magnitude of this effect was very small (Fig. 2). The functional relationship between SLA and the response was slightly curvilinear and suggested that fishing was less likely to be observed in locations with either very high positive or very low negative sea surface anomalies. Similarly, the smooth for *wind speed* indicated that fishing was less likely to be observed in areas with either very low or very high wind speeds (Fig. 3).

The effects of the variables *year*, *season* and *flag* were small but nevertheless suggested that the mean probability of fishing being observed in a location varied through time, and between the flag nationalities. Annual variation was probably explained by the differences in areas fished between years, with fishing activity most constrained in space in 2009–2010 probably due to a combination of a reduced fleet size and the influence of piracy activity on the search behaviour of vessels. Seasonal variation was probably due to differences in the geography of seasonal fishing grounds, with fishing on average more likely to be observed in any given area during the northeast monsoon months (November-April) when the fleet allocated effort over more expansive fishing grounds. Variation between flag nationalities was probably due to differences in the size of the French and Spanish fleet components, and hence the geographical dispersal of fishing activity by each respective flag in a given month.

To gain further insight into the effects of the environmental and *past use* variables on the spatial behaviour of the fleet their contribution to model accuracy was mapped in space. This was achieved by comparing for each location the accuracy of predictions generated using the AIC-best model with predictions from alternative models in which the focal variables were omitted.

**Figure 4:**
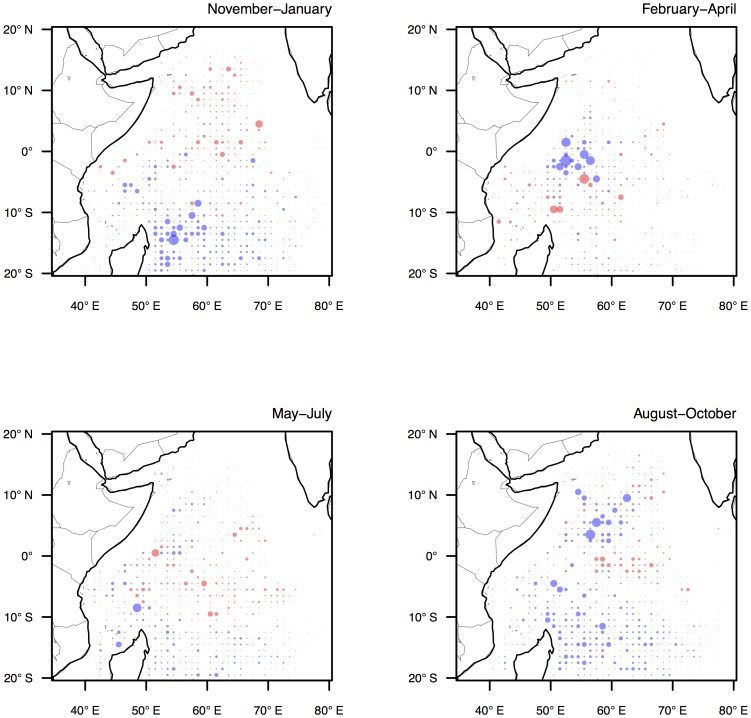
Deterioration in the accuracy of model predictions for each of the four fishing seasons when the environmental variables (*SSC, SST, SLA, wind*) were removed from the AIC-best model (*alternative 1* model). The size of the circle shows the relative magnitude of the difference in predictions and is comparable between plots. The colour indicates a more (red) or less accurate (blue) prediction.

Predictions from the *alternative 1* model, which was specified by dropping the four environmental variables (*SSC, SST, SLA, wind*) from the AIC-best model, showed both slight improvement and deterioration in accuracy in several regions. These changes in prediction accuracy tended to be correlated in space, corresponding with basin-scale gradients in environmental conditions. In all four seasons, prediction accuracy deteriorated in southern regions of the fishery, particularly below 10°S (Fig. 4). When predicting from the AIC-best model, fishing had a reasonably high probability of being observed in these areas due to the close proximity to the port of Victoria. However, these southern grounds, which are situated along the boundary of the Indian and Southern Oceans, are characterised by biologically unproductive waters, deep thermoclines and high winds, making them unsuitable fishing grounds for tropical tunas. In some seasons, particularly November-January and May-June, the omission of the environment variables in the *alternative 1* model resulted in improvements to prediction accuracy in the central and northern regions. The AIC-best model predicted a higher probability of observing fishing in these regions probably due to the relatively high levels of SSC observed. However, in actuality these regions received little fishing activity, despite having apparently suitable tuna habitat. Thus, the patterns of deterioration and improvement in prediction accuracy in the *alternative 1* model suggests that whilst environmental conditions are important in explaining the absence of fishing activity in certain areas, they are poor at predicting with certainty the presence of fishing effort.

**Figure 5:**
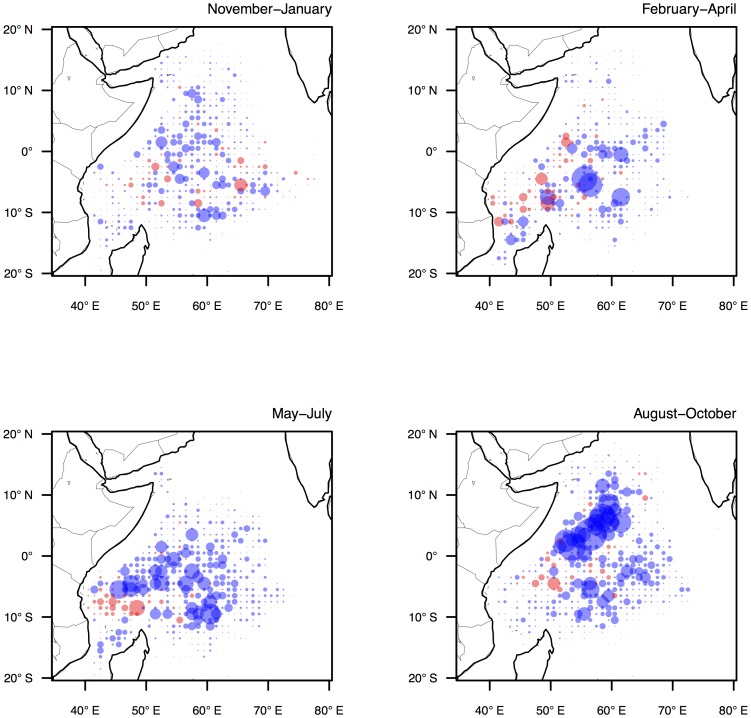
Deterioration in the accuracy of model predictions for each of the four fishing seasons when the variable *past use* was removed from the AIC-best model (*alternative 2* model). The size of the circle shows the relative magnitude of the difference in predictions and is comparable between plots. The colour indicates a more (red) or less accurate (blue) prediction.

Predictions from the *alternative 2* model, which was specified by dropping the *past use* variable from the AIC-best model, showed large deteriorations in accuracy throughout the fishery region, reiterating the importance of this variable in explaining the spatial behaviour of the fleet. Particularly large deteriorations in accuracy were evident in the Seychelles region (50–60°E) during May-July, and in the Somali Basin region (0–10°N) during August-October, which suggest habitual allocation of fishing effort into these seasonal grounds (Fig. 5). During May-July, free-swimming tuna schools are seasonally abundant in the western equatorial fishing grounds, and their surface schooling behaviour makes them especially vulnerable to purse seine gear. It is not clear to what extent this seasonal availability in the resource is coupled to environmental processes, but it appears that the location and timing of this event is well known to skippers, and this knowledge has an important influence on the spatial behaviour of the fleet. During August-October, a combination of enhanced primary productivity (reflected by high SSC concentrations) and strong ocean gyres in the Somali Basin region creates optimal conditions for fishing around floating objects (tunas tend to associate more closely with floating objects in biologically rich areas with increased forage availability; R. Bargain, skipper, pers. comm., October 2011). Whilst these fishing opportunities are more closely linked to environmental conditions, the very strong influence of the *past use* variable in explaining the presence of fishing in these relatively small grounds again implies habitual fleet behaviour.

## Discussion

The ability to anticipate the spatial behaviour of fishing fleets is of increasing importance in fishery science. On the premise that an improved understanding of effort allocation will facilitate better anticipation of fleet spatial behaviour, the aim of this study was to develop a better understanding of the factors that drive the spatial behaviour of the Indian Ocean tuna purse seine fleet.

A key finding of this study was the strong inertia observed in the spatial behaviour of the purse seine fleet, characterised by consistency in the use of seasonal fishing grounds. Patterns of effort allocation were not adequately explained by biophysical ocean conditions alone but corresponded well with past fleet behaviour, suggesting that purse seine skippers tended to fish in familiar areas in which they had some previous personal or second-hand experience (i.e. learnt from others). Also, high levels of cooperation and communication between vessels, and the long careers of many skippers (e.g.>10 years; ), have probably homogenised seasonal knowledge and experience in the fishery, which may explain the consistency in spatial behaviour at the fleet level. The indication of experience-based decision making is supported by research in the human psychology literature, which has shown that when faced with incomplete information people may not strive to make optimal decisions, but instead rely on simple heuristics (i.e. decision rules conditioned on past experience) to make decisions that achieve a satisfactory result [37], [38]. Moreover, this finding echoes those from previous studies of fisher decision making that have shown uncertainty, and associated risk, to be an important influence on fishers' expectations of catch or revenue in a location, and that familiarity can lead to habitual patterns in behaviour [25], [39]–[41].

A second important finding from this study was the bounding influence of biophysical ocean conditions on the spatial behaviour of the fleet, with certain regions characterised by unfavourable fishing conditions at any point in time and consequently not visited by the fleet. The influence of the physical environment has rarely been considered in studies of fisher behaviour, perhaps because most previous research has focused on fisheries in which the resource is associated with the sea floor (e.g. demersal trawl fisheries; [12]). By contrast, in the open ocean, the distribution of tunas and other pelagic species is influenced by biophysical conditions near the ocean surface, which can be highly dynamic in space and time [42], [43]. However, whilst these results showed that conditions associated with poor fishing conditions (e.g. cool sea surface temperatures, biologically unproductive waters) were relatively good predictors of the absence of fishing, apparently promising environmental conditions for fishing were relatively poor predictors of the presence of fishing. This result probably reflects a limitation of using biophysical ocean conditions as a proxy for the distribution of purse seine fishing opportunities, which in reality are influenced by a variety of factors. For instance, the detectability of a tuna school is influenced by the vertical distribution of a school in the water column (which can vary by species, season and region; [44]), and also the density of floating objects around which schools often associate (which can also vary by region; see [28]).

The results presented here have important management implications for anticipating the response of the purse seine fleet to events that would disrupt access to traditional fishing grounds, for example climatic anomalies, pirate activity or the implementation of spatial closures. The prediction of fleet spatial behaviour under stable fishing conditions is possible with a reasonably good level of accuracy, on the basis that strong inertia in fleet behaviour means that the distribution of effort in the past is a good predictor of where it will be allocated in the future. However, under novel conditions, such a following the closure of a significant area of fishing ground, there are likely to be considerable challenges in predicting the reallocation of fishing effort. In these situations, the past behaviour of the fleet is unlikely to be a suitable portent of where effort will be allocated, and an accurate prediction of behaviour would probably require the same near real-time information that is available to skippers themselves. Although not available in this study, information on the number and location of FADs would probably be beneficial here, particularly as skippers appear to rely on use of FADs to ‘buffer’ catches when fishing conditions are poor or when fishing grounds are closed [28]. Furthermore, the prediction of behaviour would probably need to account for the influence of group-level dynamics that emerge through knowledge sharing between skippers. The influence of processes such as teamwork and competition on the distribution of fishing effort were not sufficiently considered in this study, and neither in other similar studies, which highlights a drawback of the statistical approach used, and hence a better understanding of cooperation and competition dynamics and their influence on fleet-level behaviour are recommended as priority topics for future research.
